# The importance of continents, oceans and plate tectonics for the evolution of complex life: implications for finding extraterrestrial civilizations

**DOI:** 10.1038/s41598-024-54700-x

**Published:** 2024-04-12

**Authors:** Robert J. Stern, Taras V. Gerya

**Affiliations:** 1 Department of Sustainable Earth Systems Science, University of Texas at Dallas, Richardson, TX 75083-0688 USA; 2 Department of Earth Sciences, ETH-Zurich, Sonneggstrasse 5, 8092 Zurich, Switzerland

**Keywords:** Astrobiology, Geodynamics

## Abstract

Within the uncertainties of involved astronomical and biological parameters, the Drake Equation typically predicts that there should be many exoplanets in our galaxy hosting active, communicative civilizations (ACCs). These optimistic calculations are however not supported by evidence, which is often referred to as the Fermi Paradox. Here, we elaborate on this long-standing enigma by showing the importance of planetary tectonic style for biological evolution. We summarize growing evidence that a prolonged transition from Mesoproterozoic active single lid tectonics (1.6 to 1.0 Ga) to modern plate tectonics occurred in the Neoproterozoic Era (1.0 to 0.541 Ga), which dramatically accelerated emergence and evolution of complex species. We further suggest that both continents and oceans are required for ACCs because early evolution of simple life must happen in water but late evolution of advanced life capable of creating technology must happen on land. We resolve the Fermi Paradox (1) by adding two additional terms to the Drake Equation: f_oc_ (the fraction of habitable exoplanets with significant continents and oceans) and f_pt_ (the fraction of habitable exoplanets with significant continents and oceans that have had plate tectonics operating for at least 0.5 Ga); and (2) by demonstrating that the product of f_oc_ and f_pt_ is very small (< 0.00003–0.002). We propose that the lack of evidence for ACCs reflects the scarcity of long-lived plate tectonics and/or continents and oceans on exoplanets with primitive life.

## Introduction

A most important scientific question is whether there is life elsewhere in the universe and how to find this. We are particularly interested to find exoplanets with civilizations that can communicate with us via radio waves or other ways. Our search for habitable exoplanets is now focused on our galaxy, where we hope to find active, communicative civilizations (ACCs) among its many billion star systems. Within the uncertainties of involved astronomical and biological parameters, the Drake Equation typically predicts that there should be many (< ~100 to millions)^1^ exoplanets in our galaxy hosting ACCs. These optimistic calculations are however not supported by any significant evidence, which is often referred to as the Fermi Paradox^1^. This implies that some important variables are missing from the Drake Equation or their magnitudes are incorrectly estimated. The inconsistency has been repeatedly analyzed and various solutions have been offered to reduce the number of ACCs, in particular due to the rareness of complex life^2^ (multi-cellular life with complex cells and functions, such as algae, land plants, fungi, animals on Earth)^3–7^ compared to primitive single-cell life in our galaxy. Among others, plate tectonics has been repeatedly proposed as one of the rare conditions for complex life^2^.

Our approach to address this long-standing enigma is to re-examine the history of life on Earth. It is widely accepted that this began by 3800 Ma and that complex multicellular heterotrophs (animals) did not evolve until after 1000 Ma. Ward and Brownlee^2^ note “Over and over again the same question arises, why did it take so long for animals to emerge on planet Earth? Was it due to external environmental factors, such as the lack of oxygen for so long in the history of this planet, or to biological factors, such as the absence of key morphological or physiological innovations?” These insights build on our new understanding that the explosion of complex life (algae, land plants, animals)^3–7^ in Late Neoproterozoic time (1000–541 Ma) leading to the development of our own ACC was a consequence of a prolonged and profound transformation of Earth’s global tectonic regime from single lid to plate tectonics (“Methods”). This time period is characterized by extreme variability in atmospheric oxygen levels^7^ and records the transition from a largely bacterial toward (to a large extent) an eukaryotic phototrophic world^5,6^.

In this paper, we build on the previous studies suggesting the importance of plate tectonics for the development of complex life^2^. Firstly, we discuss the importance of the late (Neoproterozoic) onset of the modern plate tectonics regime. We begin by exploring what kinds of tectonics an active silicate body can have and then explain that such bodies likely have complex tectonic histories as they age and cool. Then we address three key aspects concerning reconstructing the evolution of the only planet with life and an ACC that we know of—Earth. Secondly, we demonstrate and explain how and why the late (Neoproterozoic) onset of the modern plate tectonics regime accelerated complex life evolution, which is the reason for us to introduce the respective f_pt_ term to the Drake equation. Thirdly, we explore for the first time why the very rare long-lasting presence of large expanses of both continents and oceans on planets with plate tectonics is an essential and restrictive condition for the evolution of ACCs, which is the reason for us to introduce the respective f_oc_ term to the Drake equation. Finally, we quantify f_pt_ and f_oc_ terms in the modified Drake Equation and show how this addresses the Fermi Paradox.

## The onset of plate tectonics in the Neoproterozoic

### On the need to consider complex tectonic histories for Earth and other active silicate bodies

Better understanding of complex physical–chemical processes associated with and possibly stimulating the major changes in life evolution^4,7^ requires detailed reconstruction, understanding and quantification of complex tectonic histories for Earth and other silicate planetary bodies. From this prospective, we discriminate two major styles of global tectonics for these bodies (“Methods”): (1) plate tectonics (PT) and (2) single lid (SL). We also propose that these bodies may experience transitions between different SL styles (e.g., Mars-style vs. Venus-style) or that PT episodes might alternate with SL episodes^8–10^. In this paper, we only go back to the beginning of Mesoproterozoic time at 1.6 Ga thereby embracing the entire period of accelerating life evolution^3,5,11^. We combine available geological indicators and their data biases (“Methods”) allowing identification of PT and SL episodes.

### What is the evidence that a transition from SL to the modern episode of PT occurred in Neoproterozoic time?

**Figure 1:**
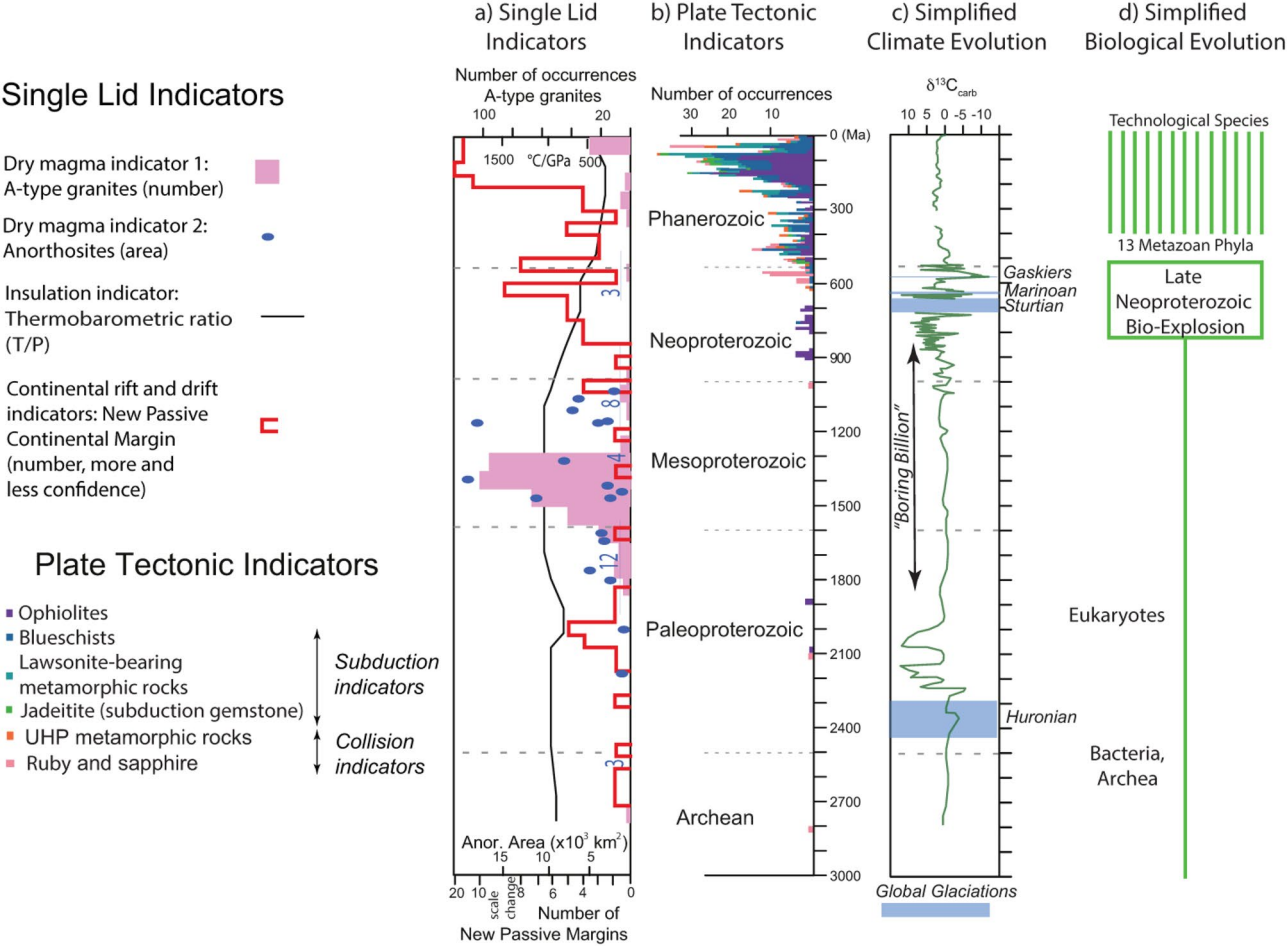
Evolution of Earth’s tectonic regime over the past 1.6 Ga. (**a**) Single lid tectonic indicators, from Stern^10^. (**b**) Plate tectonic indicators cumulative plot, modified from Stern^14^; (**c**) Simplified climate history, from Stern and Miller^28^. “Boring Billion” from Holland^37^; (**d**) Simplified biological evolution. See text for further discussion.

The time for the onset of plate tectonics on Earth remains controversial^12^. Whereas many researchers advocate that modern plate tectonic regime operated since the Archean^13^, several recent studies argue that the present regime started in the Neoproterozoic^10,14–16^, although earlier plate tectonic episodes also may have occurred^17^. The arguments also depend on what definition of plate tectonic regime (strict, or broad, “Methods”) is assumed by respective studies. Geoscientists agree that PT processes of seafloor spreading, subduction, and continental collision make distinctive minerals, rocks, and rock assemblages, called “Plate Tectonic Indicators”^14^. It should be noted, that the controversy for the onset of plate tectonics^12^ is in part related to uncertainties in interpretation of available natural data. In particular, the absence of certain PT indicators, like blueschists, may be attributed to factors such as higher mantle potential temperature and crustal composition differences^18^. Also, paleomagnetic data before 1.2 Gyr may not necessarily provide robust constraints on the continental configuration^19^. Therefore, a combined approach relying on several (rather than any single) PT indicators should be preferred^14^. Stern^14^ identified three groups of PT indicators: 1) Seafloor Spreading and Subduction Initiation Indicators; 2) Subduction Indicators; and 3) Collision Indicators (Fig. 1). These three groups of PT indicators occur overwhelmingly in Neoproterozoic and Phanerozoic time. This empirical evidence is also consistent with physical considerations built on 1) our understanding that long-lasting plate tectonics is mostly driven by the continued sinking of oceanic lithosphere in subduction zones and 2) that oceanic lithosphere was not dense and strong enough to subduct in a long-lasting continued manner as a coherent layer until mantle potential temperature cooled to less than 100–150 °C above present values^20^, which roughly correspond to Neoproterozoic mantle temperatures^21^.

The abundance of PT indicators should scale with the growth of the PT mosaic, which should take 100’s of millions of years to accomplish. Therefore, we expect the abundance of PT indicators to increase with time during the transition from SL to PT; this agrees with the Neoproterozoic record. Building on our understanding that the sinking of oceanic lithosphere in subduction zones drives plate motions, we expect that evidence of subduction initiation (Group 1 PT indicators) would appear earlier than evidence of ongoing subduction (Group 2 PT indicators); this is observed, with ophiolites appearing ~ 870 Ma and Group 2 PT indicators appearing ~ 750 Ma. Subduction would have to operate for tens of millions of years to close an ocean and cause two continents to collide. That is also observed, with Group 3 PT indicators appearing ~ 600 Ma. So the sequence of appearance of PT indicators in the Neoproterozoic – Group 1 before Group 2 before Group 3—is as expected. Another test of the hypothesis that the modern episode of PT began in Neoproterozoic time is that the preceding Mesoproterozoic Era was characterized by a SL regime. This prediction is explored in the next section.

### What is the evidence that a SL episode occurred in Mesoproterozoic time?

Given the conclusion that an active silicate body has either PT or SL (“Methods”), a requirement of the hypothesis that PT started in Neoproterozoic time is that the immediately preceding epoch – the Mesoproterozoic – was a SL episode. Stern^10^ identified three SL indicators: (1) elevated thermal regime; (2) abundance of unusual dry magmas such as A-type granites and anorthosites; and (3) paucity of new passive continental margins. Negative evidence is the lack of PT indicators. Figure 1 shows that the Mesoproterozoic was not only when few PT indicators were produced and preserved, it was characterized by abundant SL indicators. In contrast, PT indicators are documented from older Paleoproterozoic terranes^17^, suggesting adequate preservation of geologic evidence for at least the last 2 Gyr of Earth history.

A similar conclusion is reached based on types of mineral deposits, which should also be sensitive to tectonic regime. For example, orogenic gold and porphyry copper deposits, which are common in Neoproterozoic and younger times, are missing from the Mesoproterozoic^22^. In contrast, different ore types such as iron oxide copper gold (IOCG) deposits and Fe–Ti–V-P deposits associated with anorthosites are common in Mesoproterozoic terranes^23,24^.

Finally, the paleomagnetic data do not show large dispersions between continental blocks in the Mesoproterozoic, in contrast to what is documented for Neoproterozoic and younger times and for the Paleoproterozoic. In particular, the supercontinent Nuna/Columbia formed in the Paleoproterozoic and persisted through Mesoproterozoic time. For example, Evans and Mitchell^25^ noted minimal paleogeographic changes during the Mesoproterozoic. Pisarevsky et al.^26^ concluded that the supercontinent Nuna/Columbia was assembled by the beginning of Mesoproterozoic time, with some relative motion between continental blocks beginning in the mid-Mesoproterozoic.

### How long would it take for the Mesoproterozoic single lid to transform into a plate tectonic global mosaic?

**Figure 2:**
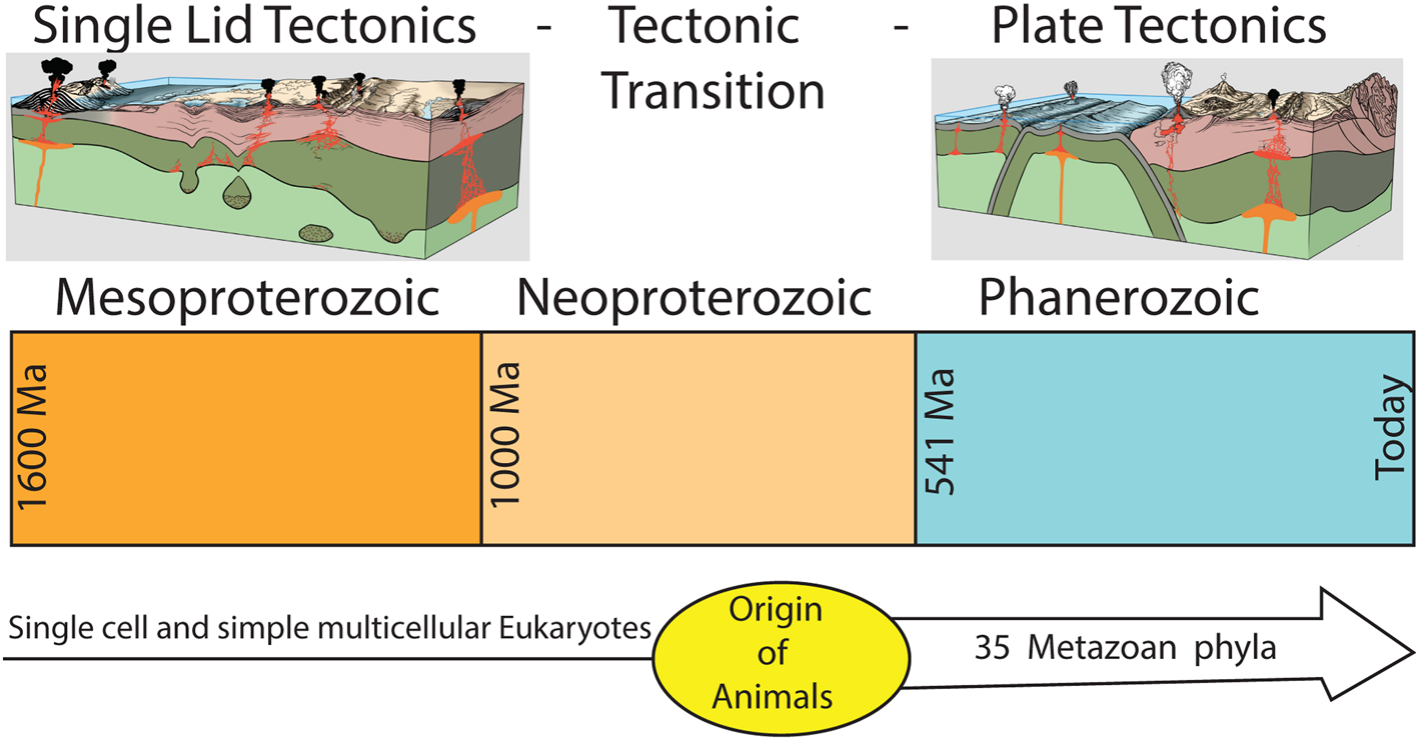
The last 1.6 Gyr of Earth’s tectonic history. See text for further discussion.

PT requires a global mosaic and this would take some hundreds million years to emerge from SL (Fig. 2). Time is required after the first subduction zone and associated transforms and divergent plate boundary form to propagate laterally and grow the mosaic. The rate of this “infection” is limited by how fast new subduction zones can form and lengthen. Trench lengthening rates accompanying Cretaceous and younger subduction initiation episodes based on observations and thermomechanical models^27^—vary from ~ 100 to ~ 600 km/Myr (100–600 mm/y). With these rates, 92 to 550 Myr would be needed to expand from a single subduction initiation point to a global plate network with ~ 55,000 km of convergent plate margins.

We can also consider the major C isotope excursions and glaciation episodes that happened in Neoproterozoic time (Fig. 1) as due to strong disruption of surface Earth systems caused by large-scale new plate boundary formation episodes. Such disturbances reflect environmental changes associated with the prolonged climate crisis called Neoproterozoic Snowball Earth. Many explanations have been offered for what caused these changes, but nearly all of these could ultimately have reflected the transition from SL to PT^28^. For example, the formation of new subduction zones and continent movements would have disrupted climate via explosive volcanism^29^ and/or true polar wander^30^. The oldest Neoproterozoic C isotope excursion is the 811 Ma Bitter Springs event^31^ and the youngest is the ~ 570 Ma Shuram event^32^, indicating a SL-PT transition that took 241 Myr. Using the first Neoproterozoic Snowball Earth glacial episode (Sturtian) which began ~ 720 Ma and the last (Gaskiers) which occurred ~ 580 Ma as marking the climate disruption due to the tectonic transition gives a slightly shorter (140 Myr) transition. C isotope excursions and evidence for glaciations in the sedimentary rock record further suggest that the tectonic transition was episodic, not smoothly continuous. It should be however noted that geochemical data also suggest the Paleoproterozoic glaciations, rise in oxygen (Great Oxidation Event, GOE) and the carbon isotope excursion at 2.5–2.05 Ga^33^, which predate the recently proposed relatively short-lived 2.05–1.8 Ga episode of plate tectonics^17^. It therefore remains partly uncertain what are the exact causal relationships between the different geochemical and climate events and the onset of plate tectonics^17,33^ ; the end of the plate tectonic episode seems to have been a result of forming the supercontinent Nuna, which terminated many subduction zones^34^. Biological consequences of this ancient PT episode also need investigation and better understanding.

## The impact of plate tectonics on biological evolution acceleration

### What is the evidence that biological evolution accelerated in Neoproterozoic time?

Life began sometime prior to ~ 3.8 Ga^35,36^. Evolution was slow for the first 3 billion years, dominated by microbes (Bacteria and Archaea), single-cell organisms that lack the membrane-bound organelles of eukaryotes, especially the nucleus, mitochondria, and chloroplasts. All complex, multicellular life is eukaryotic so single-cell eukaryotes had to evolve before multicellular algae, land plants and animals. Eukaryote fossils go back to late Paleoproterozoic time and perhaps earlier (Fig. 1). Because of the importance of oxygen to animal metabolism, multicellular animals and oxygenation of the atmosphere and ocean co-evolved. Rising oxygen concentrations due to the GOE 2.4 billion years ago facilitated eukaryotic emergence.

There are no “big events” to define when Mesoproterozoic time began and ended and what are its natural subdivisions (periods). The Mesoproterozoic Era is the heart of the “Boring Billion” (between ~ 1800 and 800 Ma; Figs. 1, 2). This term was coined by Holland^37^ because atmospheric oxygen levels did not change much during this time but now describes a protracted episode of geobiological stasis, including a remarkably stable carbon isotope record. Other indications of extended environmental stability are captured in S, Mo, Cr, Sr isotopes, and by low trace element concentrations and P in marine black shales. This protracted stable period—~ 20% of Earth history – was also a prolonged episode of low nutrient supply^38^.

The Neoproterozoic contrasts with the Mesoproterozoic by being a time of climate instability and rapidly evolving life. Neoproterozoic strata host evidence of global “Snowball Earth” glaciations, large perturbations to the carbon cycle, oceanic oxygenation, the diversification of microscopic eukaryotes, and the rise of metazoans^39^ (Figs. 1, 2). The Neoproterozoic Era is subdivided into 3 periods: the Tonian (1 Ga-720 Ma), Cryogenian (720–635 Ma) and Ediacaran (635–541 Ma). There is no question that the pace of biological evolution accelerated in Neoproterozoic time^3–5^, leading to the appearance of large multicellular organisms (or, metazoans). The much longer Tonian period was more stable, more like the Mesoproterozoic era than the much shorter and more dramatic Cryogenian and Ediacaran periods^40^. Acceleration of biological evolution characterized Cryogenian and Ediacaran time. Molecular clocks agree that animal multicellularity arose by 800 Ma (Tonian), a bilaterian body plan by 650 Ma (Cryogenian), and divergences between related phyla by 560–540 Ma (late Ediacaran)^41^. All animal phyla arose in Neoproterozoic time^42^.

### How could the Neoproterozoic tectonic transition accelerate biological evolution?

**Figure 3:**
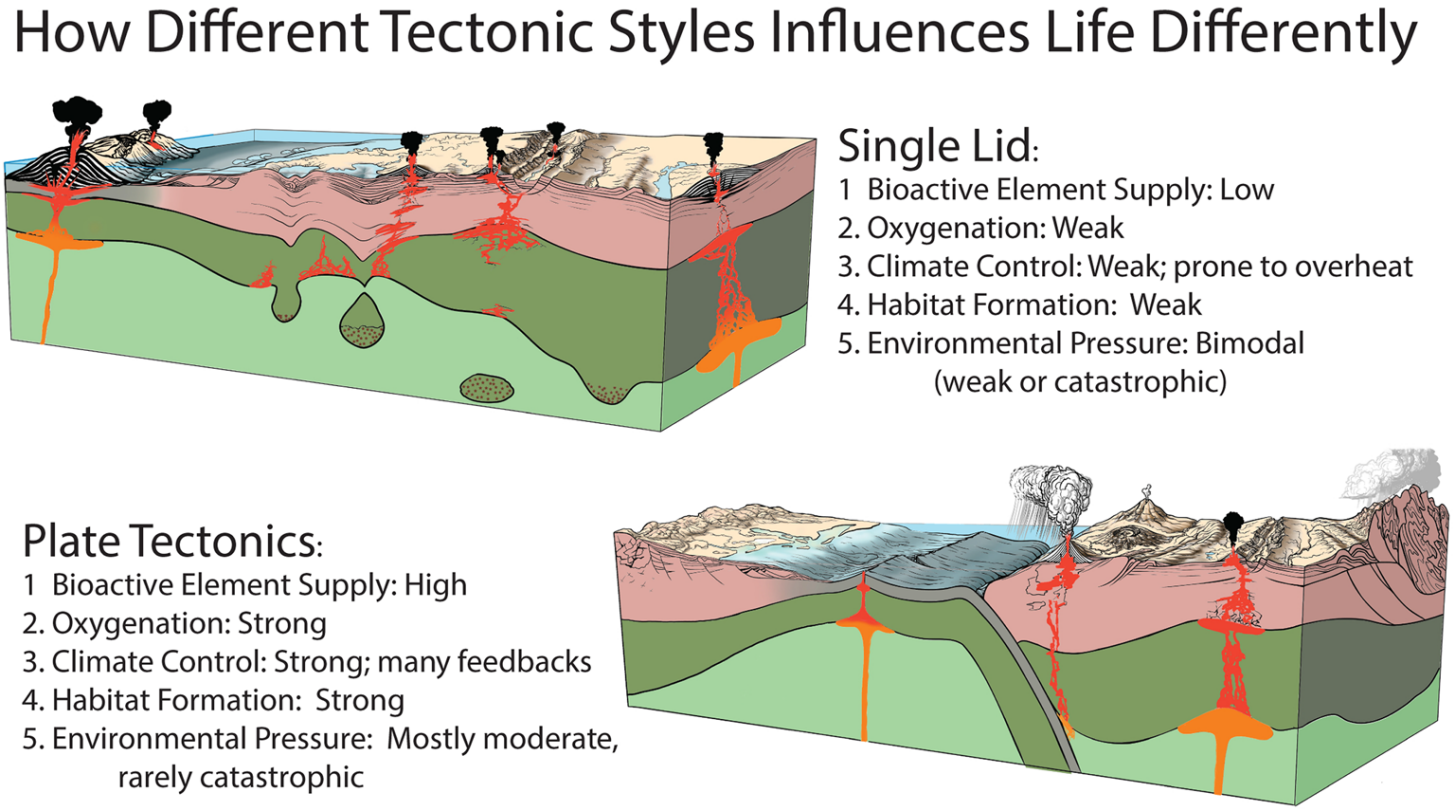
Summary diagram^43^ showing how plate tectonics stimulates life and evolution whereas a single lid tectonic style retards life and evolution. See text for further discussion.

Five processes were likely involved^43^ (Fig. 3): 1) Increased nutrient supply; 2) Increased oxygenation of atmosphere and ocean; 3) Climate amelioration; 4) Increased rate of habitat formation and destruction; and 5) Moderate, sustained pressure from incessant environmental change.

*Nutrient supply* is essential for life, especially key compounds—organic carbon, ammonium, ferrous iron and phosphate—containing C, N, Fe, and P bioactive elements respectively^44^. Phosphorus is essential because it is a globally limiting nutrient and plays a unique role in marine biogeochemistry, ecology and, hence, evolution. Researchers agree that the Mesoproterozoic biosphere was significantly less productive than today^45,46^. Because P is derived from weathering of continental crust and delivered to the ocean by rivers^47^, this suggests that decreased nutrient supply due to reduced erosion and weathering was responsible. Tectonic processes exposing fresh rocks on the surface are crucial for enhancing delivery of P and other inorganic nutrients, because shielding of fresh rock surfaces by soil reduces nutrient fluxes due to chemical weathering^48^. Rapid uplift and orogeny (Pan-African event^49^, Transgondwanan Supermountains^50^, Circum-Gondwanan Orogens^51^) at convergent plate boundaries associated with the tectonic transition would have greatly enhanced erosion, weathering and P delivery to the oceans. In this context, microbial enhancement of carbon and sulfate acid weathering^52^ acted as an important driver of nutrient delivery to the oceans from rivers. After the rise of oxygen, more weathering from more exposed land and more active bio-modulated chemical weathering^52^ resulted in enhanced erosion and enhanced burial of the organic carbon, besides delivery of P and other nutrients. As the result, P depletion of paleosols rose during the Neoproterozoic Oxidation Event (NOE) similarly to the Paleoproterozoic GOE^52^.

Support for the interpretation of unprecedented uplift, erosion, and weathering in Ediacaran time comes from the seawater Sr curve. In marine carbonates (seawater proxies), ^87^Sr/^86^Sr increased rapidly through Neoproterozoic time from near mantle-like values of ~ 0.7055 in the Tonian to the highest values in Earth history of ~ 0.7095 in early Paleozoic time^53^. Increased seawater ^87^Sr/^86^Sr reflects increased flux of radiogenic Sr from the continents, principally Pan-African uplifts, including the Transgondwanan Supermountains. Such strong uplifts require continental collision and did not occur during the Mesoproterozoic single lid episode, as shown by low marine carbonate ^87^Sr/^86^Sr record, including the ~ 1.0 Ga Grenville Orogeny^54^. The addition of P, Fe and other nutrients from erosion and weathering of Ediacaran collisional mountains broke the Mesoproterozoic nutrient drought, stimulating life and evolution. Greatly increased nutrient supply from the continents to the oceans during Neoproterozoic time is consistent with a protracted Neoproterozoic transition from Mesoproterozoic SL to Phanerozoic PT.

*Free oxygen in ocean and atmosphere* increased with time because of the proliferation of photosynthetic cyanobacteria (Fig. 1) combined with the efficient burial of organic carbon. Large, complex animals (metazoans) could not evolve during the Mesoproterozoic because they require more oxygen for respiration than was available. Minimum oxygen thresholds depend on animal size, mobility, nervous system, etc., but there is general agreement that the Mesoproterozoic atmosphere and shallow ocean contained much less than the 0.1 – 0.25 present oxygen level needed to support Cambrian metazoa^55^. A Neoproterozoic Oxygenation Event (NOE) proposed based on a range of isotopic proxies led to a much more oxygenated environment by Late Ediacaran time. There are several explanations for the NOE. One is that an increased supply of nutrients into the oceans stimulated phytoplankton growth, which converted CO_2_ into organic matter. This was further stimulated by the evolution of new plants such as algae in late Cryogenian time (659–645 Ma)^56^, which transformed the base of the food chain and produced more free oxygen. Another explanation is that enhanced chemical weathering of continents was responsible^57^. Central to all these explanations is that more dead cyanobacteria and algae – organic carbon – must be buried. Increased organic carbon burial reflected enhanced sediment supply and formation of new rift basins and passive continental margins accompanying the tectonic transition.

*Climate* is especially important for metazoans. Primitive life can exist between temperatures near the freezing of water and ~ 120 °C, but metazoans thrive between 5° and 35 °C. Plate tectonics and single lid tectonics control climate differently. PT and the supercontinent cycle controls Earth’s climate in 4 main ways. First, gases released from magmas can either warm or cool the surface, depending on their composition, which is controlled by plate tectonic setting. CO_2_ emissions associated with especially mid-ocean ridge and mantle plume igneous activity encourages atmospheric warming whereas explosive volcanism associated with convergent margins injects SO_2_ into the stratosphere to cause short-term cooling^58^. Second, proportions of Earth’s surface covered by water exert a strong control on climate, more temperate when the proportion is high and harsher when it is low. Long-term sea level rise and fall (tectono-eustasy) mostly reflects the mean age of seafloor, which changes systematically over Wilson and Supercontinent cycles. Consequently, PT Earth experienced systematic changes in climate, with warmer (greenhouse) climates about 100 m.y. after continental breakup as new oceans widen^59^. It is unknown what would control seafloor depth on SL Earth and thus how sea level would behave, but it is likely to change much less than for PT. Third, weathering of silicate rocks consumes atmospheric CO_2_ so mountain building—which exposes more silicate rocks—leads to atmospheric cooling^60^. Enhanced erosion and weathering associated with PT uplifts accompanying rifting and orogenesis releases more nutrients like P and Fe that stimulate photosynthetic life which, if sufficient dead organic C is buried, sequester CO_2_ to cool climate. Uplifts and erosion on a SL Earth should be lower so nutrient flux should be reduced and climate affected less. Fourth, subduction removes large volumes of marine carbonate rocks and organic carbon, removing CO_2_ from the near-surface and sequestering it in the mantle, leading to climate cooling^61^. Such PT controls, modified by Milankovich cycles operating over much shorter timescales^62^, are largely responsible for Earth’s climate during the time that PT has operated.

It remains debatable what are the carbon cycle and climate controls for SL planets in general^63–65^ and for Mesoproterozoic Earth in particular. The two active SL planets in our Solar System – Venus and Mars—have atmospheres that are > 95% CO_2_ thereby suggesting a reduced efficiency of CO_2_ recycling on these planets compared to Earth. On the other hand, global-scale models show that the carbon cycle could be efficiently maintained on SL planets^63,64^, where carbon recycling out of and into the mantle could occur through continuous volcanism, weathering, burial, sinking and delamination of carbonated crust. It has therefore been suggested that plate tectonics may not be required for establishing a long-term carbon cycle and maintaining a stable, habitable climate^63^. This is supported by both observations^66^ and geodynamic models^67^ showing that crustal formation and recycling also occurred throughout non-plate tectonic processes during the early Earth’s evolution, implying significant mass fluxes from the mantle to the surface and back during SL tectonic regime. In particular, the protracted Mesoproterozoic SL episode on Earth experienced a relatively stable warm climate, with no evidence for glaciation despite the Sun being ~ 5%–20% less luminous than today^68^. Elevated concentrations of greenhouse gases CO_2_ and methane (CH_4_) in the atmosphere likely kept Mesoproterozoic climate warm^69^, which needs to be reconciled with global-scale carbon cycle models^63,64^ used for planetary exploration.

*Habitat formation and destruction* is an integral part of PT via the Wilson and Supercontinent cycles governing landscape and climate evolution. Ever since Darwin visited the Galapagos in 1835, scientists have appreciated the essential role that isolated habitats play in allopatric speciation. PT makes and destroys habitats much faster and more efficiently than can active single lid tectonic regimes. The pace of evolution as a function of continental fragmentation has also been confirmed^70,71^.

*Moderate sustained pressure on organisms* from continuous environmental change happens with PT, much less so for SL. Nutrient fluxes, topography, climate, and habitats change continuously with time for PT. Strong tectonic-erosion coupling produces complex and variable landscape, climate and precipitation patterns that are especially pronounced along active plate margins. This complexity stimulates biodiversity^72^. Continental rifting and plate divergence produce large continental shelves with robust sediment and nutrient delivery from adjacent continents. The nutrients are efficiently redistributed in shelves by currents and tides, creating favorable environments for marine life^72^. All these processes were stimulated by the transition to modern PT, causing life to rapidly diversify (Fig. 3). SL tectonics is incapable of exerting moderate, sustained environmental pressure, except through the action of mantle plumes – especially when they first reach the surface and form large igneous provinces (LIPs). The most dramatic climatic effect is global warming due to increased greenhouse gases. Subsequent cooling can be caused by CO_2_ drawdown through weathering of LIP-related basalts. Other strong stresses on the biosphere include oceanic anoxia, ocean acidification, and toxic metal input^73^. It should however be mentioned that some of the feedbacks characteristic for plate tectonics may also be present during SL episodes, due to various regional-scale tectono-magmatic activities^74^ driving topographic changes and landscape and climate evolution.

### Why is the acceleration of evolution by plate tectonics critical for the development of ACCs?

Accelerated evolution of complex life by the onset of PT is critical mainly due to the general slowness of biological evolution. Timescales of biological evolution estimated on the basis of the analysis of phylogenies and/or fossils take hundreds of millions of years, comparable to timescales of major PT processes of Wilson and Supercontinent cycles^75,76^. In a constant rate birth–death model^77^, species originate with speciation rate, and become extinct with extinction rate, typically expressed as rates per lineage (L) per million years (L^−1^Myr^−1^). Estimated speciation and extinction rates typically range^76^ from 0 to 1 L^−1^Myr^v1^ and rarely exceed 1 L^−1^Myr^−1^, except during crisis intervals^75^ . This implies that during the ca. 500–1000 Myr of modern PT, only up to few hundred new complex species can have been sequentially generated (which can be truncated by species extinction) potentially (but not necessarily, due to many other possible important parameters such as reproductive style, trophic structure, social type, habitat stability, availability of resources, stability of climate, nature of predation, etc.) leading to the appearance of ACC-forming species on Earth. If one assumes this chain of new species evolution is one of the important prerequisites for the appearance of ACC-forming species on other planets, then time needed for such biological evolution will strongly depend on average effective speciation and extinction rates. We can therefore speculate that slowing speciation rates and/or increasing extinction rates (truncating evolutionary chains) by a non-stimulating global tectono-magmatic environment, such as a SL episode^78,79^, would not leave enough time for complex life to develop.

## The prerequisites of life related to oceans and continents

### Why is the presence of significant oceans and continents important for the evolution of intelligent life and civilizations?

We do not know where and how life on Earth began; furthermore with the present state of our knowledge the life origin problem cannot be solved^80^. But life existed by 3.8 Ga^35,36^ and evolved for more than 3 Gyr in the oceans. However, the presence of exposed land may also be crucial for life origin and evolution^81,82^. In particular, thermodynamic considerations indicate that the absence of exposed land would cause the total hydrolysis of all polymers and metabolites^83,84^. In addition, it has also been suggested that life originated and initially evolved in hydrated paleosols (regolith) on land rather than in the ocean^81^. On the other hand, there are at least three reasons that life evolution up to the point of metazoans must occur in water. First, seawater contains dissolved nutrients that life requires and organisms are bathed in this, making nutrients easy to absorb through initially primitive and then increasingly sophisticated evolving cell walls. Second, seawater protects organisms from deadly ultraviolet radiation; it wasn’t until about 2.4 Ga that photosynthetic cyanobacteria oxygenated Earth’s atmosphere to form a protective stratospheric ozone layer that the flux of UV radiation reaching Earth’s surface diminished significantly^85^. Third, all complex, multicellular life is eukaryotic. Single-cell eukaryotes had to evolve before multicellular plants and animals could evolve from them, and this had to happen in water because of the structural support that seawater offers. This structural support was needed for especially early metazoans, which first evolved as soft-bodied creatures in Ediacaran time. These organisms could not thrive on land until hard, strong exo- and endo-skeletons that allowed creatures to contend with gravity evolved in early Paleozoic time.

Although primitive life must evolve in the sea, advanced communicative civilizations must evolve on dry land^78^. First, changing landscape provide more varied habitats than do seascapes, and this is needed for accelerating the evolution and diversity of complex species. Consequentially, regions with high tectonic complexity, predominantly located at the confluence of major lithospheric plates such as the circum-Mediterranean, Mesoamerica, Madagascar and South East Asia, provided especially favorable sites for allopatric speciation and the emergence of new land species across straits^72^. This correlation is much less pronounced for marine species, mainly because this realm is more permeable to the movement of organisms^72,86^. Furthermore, dry land stimulated adaptations necessary for survival in harsh terrestrial environments^87^: water retention, specialized gas exchange structures, reproduction by predominantly internal fertilization, locomotion in the absence of structural support, adapted eyes and newly developed senses. These conditions stimulated development of diverse animal appendages adapted for locomotion, feeding, manipulation and other functions^88,89^, and helped to adapt eyes and other senses and the central nervous system to the new environment and functions. The resulting sophisticated bioassets allowed increasingly intelligent creatures to populate and examine the extremely variable terrestrial environments, which is one (but not the only) prerequisite to increasingly develop and transfer various experiences (i.e., knowledge and information) about these environments within biological populations. This may potentially (but not necessarily) result in the beginning of abstract thinking leading to the development of religion, science and the noosphere of Vernadsky^90^. Technology arises from the exigencies of daily living such as tool-making, agriculture, clothing, and weapons, but the pace of innovation accelerates once science evolves. Using and understanding of fire and electricity^91,92^ is essential for development of ACCs and this is unlikely in the seawater environment. On the other hand, from the planetary formation and evolution prospective, the long-term coexistence of continents and ocean on planets with plate tectonics (which are favorable for development of ACCs) is a restrictive requirement and this has to be taken into account in the Drake equation.

## Implications for the Drake equation

### The Drake equation

The Drake equation estimates how many ACCs there are in our galaxy. It is formulated as^93^:

$${\text{ACCs}}\, = \,{\text{R}}^* \, \cdot{\text{ f}}_{{\text{p}}} \cdot{\text{ n}}_{{\text{e}}} \cdot{\text{ f}}_{{\text{l}}} \cdot{\text{ f}}_{{\text{i}}} \cdot{\text{ f}}_{{\text{c}}} \cdot{\text{L}}$$

where R* = number of new stars formed per year, f_p_ = the fraction of stars with planetary systems, n_e_ = the average number of planets that could support life (habitable planets) per planetary system, f_l_ = the fraction of habitable planets that develop primitive life, f_i_ = the fraction of planets with life that evolve intelligent life and civilizations, f_c_ = the fraction of civilizations that become ACCs, L = the length of time that ACCs broadcast radio into space.

There is considerable disagreement on the values of these parameters, but the ‘educated guesses’ used by Drake and his colleagues in 1961 were:

- R* = 1/year (1 star forms per year in the galaxy)
- f_p_ = 0.2–0.5 (one fifth to one half of all stars formed will have planets)
- n_e_ = 1–5
- f_l_ = 1 (100% of planets will develop life)
- f_i_ = 1 (100% of which will develop intelligent life and civilizations)
- f_c_ = 0.1–0.2 (10–20% of civilizations become ACCs)
- L = 1000–100,000,000 years

Drake^93^ acknowledged the great uncertainties (ACCs = 200 – 50,000,000) and inferred that there were probably between 1000 and 100,000,000 ACCs in our galaxy. Other scientists used different estimates for these variables, resulting in a range of estimated ACCs from < 100 to several million^94^. The Fermi Paradox points out that all these estimates seem to be much too high. We focus on f_i_, the fraction of planets with life that evolve intelligent life and civilizations and propose to break this into two variables, f_oc_ and f_pt_, such that f_oc_ · f_pt_ = f_i_, where f_oc_ is the fraction of habitable exoplanets with significant continents and oceans and f_pt_ is the fraction of habitable exoplanets with significant continents and oceans that have had plate tectonics operating for at least 0.5 Ga.

### Why is the presence of continents and oceans unusual?

Relatively small topographic variations of terrestrial planets (< 10–20 km) suggest that the presence of both continents and oceans is restrictive in term of the required thickness (volume) and long-term stability of the surface water layer. Topographic variations are mainly driven by isostasy and are therefore expected to be nearly independent of planetary size (and surface gravity) variations. In particular, on Earth, the difference between the average elevation of continents (~ 0.835 km above sea level) and the average depth of oceans (3.7 km) is due mainly to the difference in their average vertical density profiles caused by systematic differences in their lithospheric and crustal thickness, composition and thermal structure. This imposes strict requirements in term of the optimal volume of surface water needed to satisfy stability of plate tectonics in the presence of both oceans and dry land masses. The mass fraction of surface water on Earth is 0.0224% (1 Earth ocean) and can only vary within less than one order of magnitude (from 0.007% to 0.027%, 0.3–1.2 Earth oceans) not to violate this requirement. The minimum surface water fraction (0.007%, or 0.3 Earth oceans) is given by the requirement of predominantly submarine conditions at mid-ocean ridges (average water depth 2.5 km), which is needed to produce hydrated oceanic crust that can stabilize asymmetric (one sided) subduction and plate tectonics^95,96^. On the other hand, the maximum surface water fraction (0.027%, or 1.2 Earth oceans) will flood nearly all continents without violating the condition of the presence of significant (> 5–10% of the planetary surface) land masses (such as mountain ranges, volcanic islands, rift flanks, etc.). Based on their continental freeboard model, Korenaga et al.^97^ suggested that a water world could exist on Earth with two to three oceans of water for the present and the Archean hypsometry, respectively. This could notably widen the upper bound of the permitted water mass variability to 0.045–0.067% (2–3 Earth oceans). We however prefer our more conservative upper bound 0.027% (1.2 Earth oceans) in order to ensure that significant land masses remain.

Some topographic variations are also expected with changes in planetary mass, gravity and composition^98,99^. Isostatically compensated topography should not depend on the planetary surface gravity but on lateral density changes induced by thermal and compositional variations in the crust and the mantle. The later will likely be of similar magnitude as on Earth and other terrestrial planets due to the similar nature of thermal and magmatic processes involved in crust and lithosphere formation. Some compositional variations can be expected as functions of variability in stellar and planetary compositions^98,99^, which will however remain on the same order (some hundreds kg/m^3^) as observed on Earth. Indeed, some flattening of topography with increasing planetary mass and gravity can be expected due to the incomplete isostatic compensation and lithospheric flexure effects (especially relevant for smaller planets). This may in particular further reduce permitted water mass variability on super-Earths. The expected moderate topographic variability will however likely be within the wide range of uncertainties that we will obtain by combining water mass ranges from Mars-size and Earth-size planets and the largest observed super-Earths. The water mass fraction limits should scale simultaneously in inverse proportion with the planetary radius: a Mars size planet (0.5 Earth's radius, same density) requires surface water mass fractions of 0.015–0.055% (0.1–0.3 Earth oceans), whereas the largest known super-Earth (2.35 Earth radius, same density) requires 0.003–0.012% (2–7 Earth oceans).

It has also been suggested that Earth’s mantle contains significant water and the respective mantle water mass fraction is on the order of 0.008–0.08% (0.36–3.6 Earth oceans^100^). However, the long-term stability of the surface water volume also requires stability of the water mass hosted by the mantle. This is likely caused by the mantle saturation and subsequent long-term global-scale equilibrium in the partitioning of volatiles between the interior and surface affected by the atmospheric composition, temperature and pressure^97,101–103^. The retention of water in the planetary interior could pose more restrictive conditions on the ocean formation and depth^97,103^. It should also be stressed that an addition of some stable water mass hosted in the crust and the mantle^97,101–103^ to the mass of the surface ocean will not widen the range of permitted variability of the total water mass delivered to a planet, which would allow the long-term coexistence of continents, oceans and plate tectonics.

Mass-balance calculations indicate that a cometary contribution to Earth’s water was probably limited to $\le$ 1%^104^. On the other hand, meteorite data and planetary formation models suggest that some variable amount of water can be delivered to relatively dry terrestrial planets by water-rich planetesimals formed in the outer Solar System beyond the “planetary snowline” and scattered inwards during the growth, migration, and dynamic evolution of the giant planets^105,106^. In such planetary formation scenarios, the amount of the delivered water can be highly variable^105^. Assuming 0–90% volatile lost during impacts^105,107^, and 5–10% water content in the water-rich planetesimals total delivered water mass fraction can range from 0.008–3.8%^105^, which is a much broader range than the required optimal water mass variability. The potential planetary water mass fraction variability can be further broadened by considering the possible existence of ocean worlds^108^ (such as Europa and Callisto, mass water fraction 6–55%^109^). The expected large variability of planetary water mass fractions (0–55%) makes the requirement of the long-tem existence of the optimal surface water volume to be a kind of “Goldilocks condition”. By comparing the permitted variability ranges for planets of different size (0.009–0.04%) and the expected variability due to different planetary accretion scenarios (up to 3.8–55%) we can evaluate f_oc_ as the probability for a planet to have the optimal surface water volume to be on the order of 0.00016 – 0.011. This probability range can be tested by using the recent work of Kimura and Ikoma^110^, which predicted diversity in water content of terrestrial exoplanets orbiting M dwarfs. Based on the available data from their simulations^110^, the results for planets with 0.5–2.35 Earth radii suggest variability of water mass fraction to range from 0–56%. The fraction of the planets with the optimal water volume is very small and ranges from f_oc_ = 0.0006–0.0022, which is thus well within the range of our estimate.

### Why plate tectonics operating for at least 0.5 Gyr is unusual

Plate tectonics is unique to Earth and no other terrestrial planet or satellite in the Solar system has plate tectonics, although episodic regional-scale subduction processes have been identified on Venus^111,112^. The presence and sufficient depth of the surface ocean seems to be necessary to ensure the long-term stability of continued subduction^95,96^, and is already included in our f_oc_ estimate. An important additional restriction comes from the stellar composition. Unterborn et al.^98^ found that only 1/3 of the range of stellar compositions observed in our galaxy is likely to host planets able of sustaining density-driven tectonics (such as plate tectonics), which sets an upper limit of f_pt_ to 0.33. This relatively strong reduction, does not however take into account possible devolatilization effect for estimating rocky exoplanet compositions^99^. Spargaren et al.^99^ have recently quantified these effects and obtained notably modified planetary compositions compared to earlier works^98^. They, however pointed out that their obtained planetary compositions rich in Na and Si will have more buoyant crusts than Earth, which may render subduction and hence plate tectonics less efficient^98,99^. Therefore, in the absence of any other estimates of the likelihood of sustaining density-driven tectonics on exoplanets, the value proposed by Unterborn et al.^98^ will have to serve for our estimation of f_pt_.

Further reduction of f_pt_ may come from the consideration that relatively small terrestrial planets such as Mars or Mercury should have strongly lowered convection vigor due to their low gravity and smaller mantle thickness, which make them unlikely candidates for the long-term stability of a plate tectonic regime. Planetary accretion models^113^ show that the fraction of such small terrestrial planets can be large (ca. 50%), which implies respective reduction of f_pt_ to 0.17. It is also not clear if plate tectonics is more likely or less likely^114–117^ on large terrestrial planets (super-Earths). Therefore, f_pt_ may have to be further reduced to exclude super-Earths, which are common in our galaxy^113^. Another restriction may come from the fact that continued (rather than intermittent) subduction requires a limited range of mantle potential temperatures^20^, which can only be realized in part of the planetary cooling history^118^. In the case when planetary evolution starts from cooler mantle temperature than Earth, plate tectonics may never start and single lid tectonics may operate for the entire planetary history^118^. This should thus further reduce the f_pt_ value to exclude planets with insufficiently hot mantles during their evolution. Unfortunately, this reduction cannot be easily quantified and simply implies that f_pt_ < 0.17.

### Possible solution to the Fermi Paradox

Based on the modified Drake Equation, we suggest that the Fermi Paradox may be resolved if the product of f_oc_ and f_pt_ is very small. Our preliminary estimates show that f_oc_ can be on the order of 0.0002 – 0.01 whereas f_PT_ is < 0.17, which makes their product f_oc_ · f_pt_ = f_i_ to be extremely small (< 0.00003 – < 0.002). This estimate drastically reduces the potential number of ACCs (to < 0.006 – < 100,000) in our galaxy calculated with the modified Drake equation. Further significant reduction may come from the re-evaluation of the characteristic length of time for ACCs communication activities (L). Values of L can be limited to 400–7,800,000 years by societal collapse^119^ and biological species survival^120^, which again reduces the potential number of ACCs in our galaxy to even lower numbers (< 0.0004 – < 20,000). The value less than 1 of the lower bound implies that the probability to find at least one ACC (including ourselves) in our galaxy can be as low as < 0.04% (this lower limit is however strongly dependent on the large remaining uncertainties of parameters in our modified Drake equation). As the result, it may be that primitive life is quite common in the galaxy. However, due to the extreme rareness of long-term (several hundred of million years) coexistence of continents, oceans and plate tectonics on planets with life, ACCs may be very rare.

On the other hand, the chances of finding planets with life, continents oceans and plate tectonics (i.e., COPT planets) in our galaxy, which are potentially suitable for ACCs, by remote sensing are relatively high. They can be evaluated on the basis of the Drake equation modified for the purpose of remote sensing as

$${\text{COPTs}} = {\text{ R}}^* \, \cdot{\text{ f}}_{{\text{p}}} \cdot{\text{ n}}_{{\text{e}}} \cdot{\text{ f}}_{{\text{l}}} \cdot{\text{ f}}_{{{\text{oc}}}} \cdot{\text{ f}}_{{{\text{pt}}}} \cdot{\text{ L}}_{{{\text{COPT}}}}$$

where: L_COPT_ = 500,000,000 yr is the characteristic time of the long-term coexistence of ocean continents and plate tectonics in Earth history needed for the accelerated development of advanced life. The resulting expected number of COPT planets in our galaxy ranges between 500 and ca. 1,000,000, which create reasonable chances of finding them by future exoplanetary exploration.

## Methods

### Single lid vs. plate tectonics

Based on the presence/absence of an active global plate mosaic^121^, silicate planetary bodies of the Solar System show two major types of tectonics: plate tectonics (PT, the global plate mosaic is present, modern Earth) and single lid (SL, the global mosaic is absent, Mars, Moon, Io, Venus, perhaps Archean-Hadean Earth). SL behavior can be further subdivided into three main sub-types characteristic for different planetary bodies depending on their size and interior temperature. From most convectively active to dead silicate bodies, these include: (1) volcanic heat pipe (small bodies with hot interior, Io), (2) squishy lid (large bodies with hot interior, Venus, Archean-Hadean Earth^122^); (3) stagnant lid (small bodies with little convection, Mercury, Mars). We use a strict definition of PT, which requires the presence of a global plate mosaic driven by long-lasting subduction^123^. This allows discrimination of modern Earth’s tectonic regime from other types of mobile planetary surface behavior (like Venus)^111,112,124^, in which (i) localized plate boundaries do not exist or do not form a global plate mosaic and (ii) horizontal surface motions are not predominantly driven by oceanic plate subduction (we classify this surface behavior as single lid tectonics^125^). Three out of four actively convecting silicate bodies in our Solar System have SL tectonics, so this is likely to dominate the tectonic styles of active silicate bodies in our galaxy. Because PT does not occur on any other planet, it may be that the PT regime is also unusual in Earth’s tectonic history.

### Is the geologic record too biased to reconstruct Earth’s tectonic history?

The geologic record is biased to younger rocks^126^ but how far back in time is the record good enough to allow Earth’s tectonic history to be reconstructed? Some argue that it is too incomplete for Earth’s tectonic history to be reconstructed far into the Precambrian. There is strong evidence that deep erosion in Late Neoproterozoic time to cut the Great Unconformity, removing 3–5 km of rock, was caused by extensive glaciation^127^. Such deep erosion could have removed older group 1 PT indicators (ophiolites) but this does not seem to have happened because early Neoproterozoic (Tonian) ophiolites are well preserved^128^. Also, several well-preserved ophiolites that formed 1.9–2.1 Ga are known (Fig. 1), indicating that preservation is good enough and suggesting that an episode of proto-PT occurred in Paleoproterozoic time^17^. One occurrence of 3.8 Ga ophiolites^129^ may suggest viability of oceanic spreading and episodic regional subduction in squishy-lid Archean Earth in agreement with recent numerical models^74^. Even if some evidence from supracrustal rocks like ophiolites has been removed, deep erosion would not remove groups 2 and 3 of PT indicators, which are metamorphic rocks and exist deep in the crust. On the basis of these arguments, we think that Earth’s tectonic history can be reconstructed back to at least 2.5 Ga, the beginning of Proterozoic time.

## Data Availability

We analyzed results of Monte Carlo simulations of Kimura and Ikoma^110^, which are publically available via GitHub at https://github.com/TadahiroKimura/Kimura-Ikoma2022.
